# Role of Macrophages in Cardioprotection

**DOI:** 10.3390/ijms20102474

**Published:** 2019-05-19

**Authors:** Jonathan Yap, Hector A. Cabrera-Fuentes, Jason Irei, Derek J. Hausenloy, William A. Boisvert

**Affiliations:** 1 Center for Cardiovascular Research, John A. Burns School of Medicine, University of Hawaii, Honolulu, HI 96813, USA; jktyap@hawaii.edu (J.Y.); jasonsi@hawaii.edu (J.I.); 2 Tecnologico de Monterrey, Centro de Biotecnologia-FEMSA, Monterrey, NL 264610, Mexico; 3 National Heart Research Institute Singapore, National Heart Centre, Singapore 169609, Singapore; d.hausenloy@ucl.ac.uk; 4 Cardiovascular and Metabolic Disorders Program, Duke-National University of Singapore Medical School, Singapore 169857, Singapore; 5 Institute of Biochemistry, Medical School, Justus-Liebig University, 35392 Giessen, Germany; 6 Institute of Fundamental Medicine and Biology, Kazan (Volga Region) Federal University, 420008 Kazan, Russia; 7 Yong Loo Lin School of Medicine, National University Singapore, Singapore 117597, Singapore; 8 The Hatter Cardiovascular Institute, University College London, London WC1E 6HX, UK; 9 The National Institute of Health Research University College London Hospitals Biomedical Research Centre, Research & Development, London W1T 7DN, UK

**Keywords:** macrophages, cardioprotection, innate immune response, myocardial infarction, cardiac repair, remodeling

## Abstract

Cardiovascular diseases are the leading cause of mortality worldwide. It is widely known that non-resolving inflammation results in atherosclerotic conditions, which are responsible for a host of downstream pathologies including thrombosis, myocardial infarction (MI), and neurovascular events. Macrophages, as part of the innate immune response, are among the most important cell types in every stage of atherosclerosis. In this review we discuss the principles governing macrophage function in the healthy and infarcted heart. More specifically, how cardiac macrophages participate in myocardial infarction as well as cardiac repair and remodeling. The intricate balance between phenotypically heterogeneous populations of macrophages in the heart have profound and highly orchestrated effects during different phases of myocardial infarction. In the early “inflammatory” stage of MI, resident cardiac macrophages are replaced by classically activated macrophages derived from the bone marrow and spleen. And while the macrophage population shifts towards an alternatively activated phenotype, the inflammatory response subsides giving way to the “reparative/proliferative” phase. Lastly, we describe the therapeutic potential of cardiac macrophages in the context of cell-mediated cardio-protection. Promising results demonstrate innovative concepts; one employing a subset of yolk sac-derived, cardiac macrophages that have complete restorative capacity in the injured myocardium of neonatal mice, and in another example, post-conditioning of cardiac macrophages with cardiosphere-derived cells significantly improved patient’s post-MI diagnoses.

## 1. Cardiovascular Disease and Global Burden 

Cardiovascular diseases (CVD) are the leading cause of death and disability worldwide [1]. CVD are a class of diseases which affect the heart, blood vessels, and the vasculature of the brain [1]. CVD include but are not limited to atherosclerosis, heart disease, ischemic heart disease, cerebrovascular disease, ischemic stroke, hemorrhagic stroke, hypertensive heart disease, cardiomyopathy and myocarditis, atrial fibrillation and flutter, aortic aneurysm, peripheral vascular disease, and endocarditis [2]. According to 2015 mortality data, CVD claimed an estimated 17.9 million deaths globally, more than cancer and chronic lower respiratory disease combined [3]. Disability adjusted life-years (DALYs), is a measurement for the number of years lost due to illness, disability, and early death. In 2016, global CVD burden was estimated to be 353 million DALYs compared to 308 million DALYs in 2000 [4].

## 2. Overview of Atherosclerosis

Atherosclerosis is an inflammatory disease characterized by the hardening and narrowing of an artery due to the accumulation of lipids, immune cells, ribonucleic acids and various fibrous elements [5,6,7,8]. Over time these accumulations develop into atherosclerotic plaques that can occlude blood vessels resulting in reduced blood flow and may lead to acute thrombotic complications [9,10,11,12,13,14]. Thrombotic events arise when vulnerable plaques rupture, exposing vascular structures to circulation setting off a coagulation cascade forming a thrombus [12,15]. This thrombus may either form a stationary blockage completely occluding the blood vessel leading to symptoms of acute ischemia, or break loose from the initial formation site and become lodged elsewhere potentially resulting in myocardial infarction (MI), pulmonary embolism (PE), or stroke [12,16,17,18,19,20,21]. As extensively described in this review, macrophages play a central role in stoking inflammation in the cardiovascular network. Recent clinical studies aimed at attenuating inflammation in CVD through inhibition of IL-1β is a powerful testament to the importance of inflammation in CVD [22,23].

## 3. Macrophage Roles in Thrombus Formation

Early development and progression of atherosclerosis predominantly occurs within arterial areas which experience disturbed laminar flow such as arterial branch points and bifurcations [24]. In these areas, low-density lipoprotein (LDL) and apolipoprotein B-containing lipoproteins accumulate within the sub endothelial space where they are subject to modification by reactive oxygen species (ROS) and various enzymes [11,24,25,26,27]. Monocytes infiltrate the vascular intima, differentiate into macrophages, and phagocytize modified LDL within the surrounding tissues. Macrophages are unable to regulate metabolism of modified lipid species and become lipid-laden foam cells leading to dysregulation of inflammatory signaling, endoplasmic reticulum (ER) stress, and eventually cell death [28,29,30,31,32,33]. Proliferation of smooth muscle cells assist in the formation of a fibrous cap on the luminal side of the plaque, contributing to plaque stability [34]. As atherosclerosis progresses, disruption of macrophage-mediated efferocytosis fails to effectively clear cellular debris and oxidized lipids, leading to the development of a necrotic core within the plaque [24,35,36]. Metabolically dysfunctional macrophages together with necrosis, release proteolytic enzymes capable of thinning the protective fibrous cap [24,37,38]. Matrix metalloproteinases (MMPs) produced by macrophages can degrade various types of extracellular matrix (ECM) proteins [24]. MMP-2 and MMP-9 are hypothesized to play a role in fibrous cap thinning and plaque rupture [13,24,39]. Rupture of an atherosclerotic plaque may lead to life threatening conditions such as a myocardial infarction, stroke, pulmonary embolism, and limb ischemia [12,19,21,40,41]. Recent evidence has demonstrated that macrophage interactions with platelet-derived chemokines play a crucial role in atherothrombotic risk. CXCL4 is released from platelet alpha granules which stimulates the release of other store proteins including the chemotactic cytokines; CCL3 (MIP-1α), CCL5 (RANTES), and CCL7 (MCP-3) [42,43,44]. Transcriptomics revealed that CXCL4 induces a novel alternatively activated macrophage termed “M4”, and data has also shown that M4 macrophages are significantly associated with atherosclerotic plaque stability and vascular inflammation [45,46,47].

While the majority of MI events occur as a function of fibrous cap rupture due to inflammatory and proteolytic degradation by macrophages and constituents of the necrotic core, another phenotype of MI is responsible for nearly 30% of all thrombotic events and results from plaque erosion and thrombus formation without rupture of the fibrous cap [48,49]. And although macrophages have long been accepted as the purveyor of MI, studies have shown that plaque erosion is associated with less lipid/plaque burden, a greater abundance of smooth muscle cells, and a comparatively small macrophage population [50,51,52,53]. Consequences of such limited macrophage content associated with plaque erosion in contrast to macrophage-dependent plaque rupture have yet to be determined, and the mechanism of erosion vs. rupture requires further exploration.

## 4. Macrophages: Inflammation and Activation

As the macrophage is the predominant cell type in all stages of atherosclerosis, phenotypic heterogeneity and functional plasticity are fundamental to a given inflammatory response. Resolving inflammation is a regulated inflammatory process which relies on immune cells to maintain systemic homeostasis [54]. This is accomplished by immediate elimination of foreign pathogens, resolution of sterile inflammation by removal of endogenous pathogens, and maintenance of proper wound healing and tissue repair [55]. Following the clearance of inflammatory elements, most macrophages leave the inflammatory site or undergo apoptosis [56]. In contrast, non-resolving inflammation is chronic in nature as its underlying cause persists for an extended period of time [57]. Cancer, arthritis, and atherosclerosis are all examples of non-resolving inflammation in which infiltration and accumulation of monocytes, dendritic cells, and macrophages induce a continuous inflammatory cascade [58,59,60]. The physiological environment is responsible for generating different subtypes of macrophages that display varying degrees of activation. Macrophage activation produces distinct functional phenotypes that are most commonly categorized using the Th1/Th2 T cell polarization paradigm, whereby M1 macrophages are representative of pro-inflammatory characteristics and M2 macrophages are associated with tissue repair and wound healing [61,62]. Clearly, this is an overly simplistic view of macrophage behavior. However, the general guidelines that advise the M1 and M2 nomenclature are useful as a basic delineation of macrophage phenotypes.

M1 macrophages are described as being “classically” activated. M1 activation of macrophages can be initiated by recognition of pathogen-associated molecular patterns (PAMPs) like lipopolysaccharide, chitin, and other intracellular pathogens. In addition, damage-associated molecular patterns (DAMPs) resulting from tissue/cell damage and necrosis are also capable of promoting inflammation. Typically, PAMPs and DAMPs bind to pattern recognition receptors (PRRs) located on the surface of macrophages. Toll -like receptors (TLRs) are one of five types of PRRs associated with macrophage activation [63,64,65,66]. During an innate immune response, TLR agonists engage the MYD88-dependent pathway including IRAK4, TRAF6, and IKKβ ultimately leading to activation of NF-κB. The inflammatory function of this pathway in M1 macrophages is essential to innate immunity in response to pathogenic stimuli. NF-κB activity in M1 macrophages results in transcription of pro-inflammatory cytokines: IL-1B, IL-6, IL-12, TNF-α, as well as chemokines involved in immune cell recruitment and invasion. M1 macrophages also encourage differentiation of inflammatory T-cell phenotypes which further participate in mediating inflammation [67,68]. IFN-γ is also known to promote M1 activation. It is a potent microbicidal, cytotoxic effector that is transiently produced by natural-killer cells (NK) and sustained production is achieved by Th-1 cells. IFN-γ relies on the JAK/STAT signaling pathway which is initiated by receptor-ligand interaction with IFN-γ receptor 1 and 2 (IFNGR1/2) [69]. It has recently been shown that IFN-γ and TLR mediated signaling pathways have a synergistic effect on promoting the M1 macrophage phenotype with tumoricidal function. The resultant macrophage population produces nitric oxide (NO), TNF-α, IL-12p40, and IL-12p70 while the presence of IFN-γ suppressed macrophage secretion of IL-10 is induced by TLR agonists [70]. During the innate immune response, classically activated macrophages produce pro-inflammatory cytokines which play a key role in host defense. However, prolonged exposure to such compounds can result in extensive damage to the host. This is the case in various inflammatory diseases [71,72,73].

Alternatively activated macrophages (M2) are characterized as having proliferative and wound healing properties. M2 macrophages promote collagen synthesis, fibrosis, and other tissue remodeling functions [74]. Further, this macrophage phenotype displays elevated expression of scavenger receptors (SR), mannose receptors (MR), and galactose receptors. Alternatively activated macrophages secrete cytokines: Transforming growth factor-β (TGF-β) and IL-10, chemokines: CCL17, CCL22, CCL24, and the enzyme arginase-1 in mice (ARG 1), which participates in cell proliferation, collagen formation, and tissue repair [74,75,76]. M2 polarization of macrophages is commonly accomplished by stimulation of the glucocorticoid receptor (GCR), IL-10 receptor (IL-10R1), and antigens recognized by the Fc family of receptors [76]. IL-4 and IL-13-mediated alternative activation represents the most extensively studied variation of M2-macrophage plasticity. Both of these Th 2-related cytokines are expressed by T cells, B cells, mast cells, and macrophages. Aside from down regulating many of the cytokines associated with M1 activation, IL-4 and IL-13 induce fibrogenesis through expression of fibronectin-1 (FN-1) and beta 2 integrins. They also modulate tissue repair and cell proliferation signaling via insulin-like growth factor 1 (IGF-1) [77,78]. Aberrant expression of IL-4 and IL-13 has been associated with fibrosis in different disease states and tissues [79,80]. The term “alternative activation” refers to any activation state other than “classically” activated. Therefore, it should be noted that alternatively activated macrophages exhibit a growing spectrum of phenotypic and functional varieties. M2 macrophages can be induced by different stimuli as previously mentioned, and accordingly have specific classifications denoted by the stimulus and effector function. M2a macrophages are elicited by Th-2 cytokines, IL-4 and IL-13 while M2b activation is triggered by Fc-γ receptors and TLR stimulation, and M2c macrophages can be obtained by GC, IL-10, or TGF-β ligands [81]. 

## 5. Cardiac Macrophages

Tissue-resident macrophages have been identified in a number of different organs where they engage in maintaining homeostatic conditions and tissue regeneration. Mouse models have demonstrated that tissue-resident macrophages found in the liver, brain, lung, skin, and heart derive from an embryonic lineage, distinct from those of monocytic progenitors [82,83,84,85,86,87]. These studies and others also highlight the observation that tissue-resident macrophages maintain a self-proliferative population throughout adulthood, which is independent of monocyte-derived macrophage recruitment and declines with age [88,89,90]. The majority of resident cardiac macrophages were found to be established prior to birth, and yolk sac progenitors are the primary source of this cell population [91]. Essentially, the development of advanced gene fate-mapping techniques has shown that, in the steady-state, two resident cardiac macrophage subsets are present: MHC-IIlowCCR2- and MHC-IIhighCCR2- cells. Resident cardiac macrophages replenish through tissue proliferation, and comprise the largest subpopulation of cardiac macrophages [91,92]. Under inflammatory conditions, a third macrophage subtype can be found in the heart and is classified as MHC-IIhighCCR2+ cells. Originating completely from bone marrow-derived monocytes, the population of this macrophage subtype is recruited during inflammation and ultimately replaces embryo-derived cardiac macrophages as their proliferative properties diminish with age [91,93]. Circulating CCR2+ monocytes interact with the CCR2 ligand, MCP-1 (CCL2), which is a chemotactic cytokine that potentiates macrophage recruitment and invasion [94]. Cardiac tissue injury initiates an influx of CCR2+ macrophages which produce IL-1β, pro-inflammatory cytokine associated with atherosclerosis [22,91]. On the other hand, resident CCR2- macrophages primarily originating from an embryonic progenitor facilitate processes of angiogenesis and cardiomyocytes proliferation, roles that are similar in developmental and neonatal macrophages [95,96]. Bajpai et al. demonstrated that the depletion of resident cardiac CCR2- macrophages in a murine model of myocardial infarction increased infarct area, reduced left ventricular (LV) systolic function, and exaggerated LV remodeling [97]. Another study investigated the therapeutic potential of resident cardiac macrophages by administering a selective CCR2 inhibitor in a mouse model of cardiac injury. CCR2 inhibition blocked monocyte recruitment and preserved the populations of MHC-IIlowCCR2- and MHC-IIhighCCR2- resident cardiac macrophage subsets. As a result, decreased mRNA and protein expression of MCP-1, IL-1-β, IL-6, and TNF-α was observed in injured mice that received the CCR2 inhibitor compared to vehicle control [96]. Further exploration into resident cardiac macrophages as a target for improved outcomes after myocardial infarction is indeed warranted.

Monocyte-derived macrophages are characterized as MHC-IIhighCCR2+ cells which are highly recruited to the heart under atherosclerotic conditions and following myocardial insult. As atherosclerosis develops and progresses, monocytes are recruited to lesions where they accumulate and contribute to atheromatous plaque formation. Unlike Ly-6C^Low^ monocytes which are far less invasive, Ly-6C^High^ monocytes induce excessive monocytosis, accumulate in lesions, and differentiate into macrophages [98,99]. Ly-6C^High^ Monocytes are inflammatory and rely on CCR2, CCR5, and CX3CR1 to infiltrate plaque sites [100]. Further, Combadière et al. found that inhibition of CCL2, CCR5, and CX3CR1 abrogated monocytosis and trafficking of Ly-6CHigh monocytes, which abolished atherosclerosis by up to 90% in hypercholesterolemic mice [101].

## 6. Macrophages in Homeostasis

In the healthy heart, mouse models reveal the presence of a significant number of resident cardiac, yolk sac-derived MHC-IIlowCCR2- and MHC-IIhighCCR2- macrophages expressing low levels of Ly-6C and exhibiting an M2 polarization profile [102]. It has recently been demonstrated that local proliferation of these resident tissue macrophages is the major source of cardiac macrophages in the steady-state [91,103]. Data published by Epelman et al. utilized the proliferation marker Ki-67 to demonstrate the rapid proliferation of resident cardiac macrophages in the heart after MI, while proliferative activity is completely ablated in monocytes recruited from the circulation once they have differentiated into macrophages [91]. Conversely, experimental mouse models have revealed that circulating monocytes contribute to inflammatory mechanisms that precede different forms of cardiac pathology. SiRNA silencing of CCR2 mRNA in monocytes prevented monocyte accumulation in inflammatory regions and in atherosclerotic plaques, and reduced infarct size after coronary artery occlusion [104]. Resident cardiac MHC-IIlowCCR2- and MHC-IIhighCCR2- macrophages express high levels of myeloid epithelial reproductive tyrosine kinase (MerTK), a critical receptor involved in macrophage efferocytosis. Deficiency of this protein results in inefficient removal of cell debris during cardiac repair, increased infarct size, and depressed cardiac function [105]. 

## 7. Macrophages in the Aging Heart

As is the case with most diseases and disorders, age is a major risk factor in cardiac-related morbidity and mortality. Aging in the heart is often characterized by a number of dysfunctional conditions including: Myocardial sarcopenia, hypertrophy, vascular hyperpermeability, fibrosis, inflammation, and functional impairment [106,107,108]. Direct changes in the myocardium and not ventricular load as a result of hypertension is responsible for such alterations in heart condition and function [109]. Employing genetic fate-mapping techniques along with parabiotic and chimeric bone marrow mouse models, Molawi et al. essentially demonstrated that the self-renewal of yolk sac-derived resident macrophages declines with age. Thus, population dynamics in the aging heart shifts in favor of monocyte-derived macrophages even in the absence of inflammation [93]. These examples represent a collective body of data that grows more compelling with the assertion that resident cardiac macrophages hold much potential in the way of therapeutic intervention for a wide array of cardiac disorders.

## 8. Pathology and Resolution of Myocardial Infarction

Myocardial infarction (MI) is one of many pathological etiologies associated with cardiovascular disease. In the pathologic context MI is defined as cardiomyocyte death due to ischemic insult. Such events occur as a function of an imbalance in the relation between myocardial oxygen supply and demand. The continuous development of atherosclerotic plaque in the vasculature results in occlusion of the vessel lumen. Luminal narrowing of ≥75% can go undetected while at rest, but as oxygen demands increase with activity, the obstructed blood flow does not allow for proper oxygenation of tissues. The most common underlying cause of MI is the incidence of plaque instability in atherosclerotic disease [110]. Stability in the atheromatous plaque is dependent on the structural integrity of the fibrous cap. Plaque rupture or erosion introduces thrombi into the circulation where they can cause thrombotic embolism and acute MI [111]. Occlusion of coronary blood flow prevents aerobic metabolism leading to rapid ATP depletion and metabolite accumulation. Critical systolic dysfunction is observed within seconds of ischemic insult and has been shown to cease entirely after 60 s [112,113]. The effects of ischemic insult are completely reversible so long as the duration is <15–20 min. In large animal models, prolonged periods of ischemia (60 Minutes) causes continuous cell death of cardiomyocytes in the ischemic region [114,115,116]. After a significant MI, cardiomyocytes undergo extensive cell death. The mammalian heart has only limited regenerative capacity, and therefore the large number of deceased cells are replaced by non-contractile scar tissue derived from collagen and ECM production. Although this scar tissue is largely nonfunctional, it is crucial to the maintenance of cardiac structural integrity and organ function. This reparative process following MI generally takes place in a two-tiered fashion; an inflammatory stage in which a massive influx of immune cells are recruited to the ischemic region where macrophages and neutrophils mediate efflux of necrotic debris, and a reparative/proliferative phase that involves the release of growth factors accompanied by extracellular matrix formation, collagen synthesis, and scarring [117,118,119,120,121]. Dysregulation of the inflammatory response associated with severe MI can result in adverse remodeling, increased risk of repeat events, and pervasive fibrosis that ultimately compromises cardiac function [122].

## 9. Macrophages in Myocardial Infarction

As previously mentioned, cardiac macrophages are of two distinct lineages: Yolk sac-derived MHC-IIlowCCR2- and MHC-IIhighCCR2- macrophages and MHC-IIhighCCR2+ monocyte-derived macrophages. Over time, resident developmental macrophages are progressively replaced by macrophages differentiated from bone marrow-derived and splenic monocytes. Macrophages are among the most abundant cell types in the infarcted heart. Circulating monocytes from the bone marrow and spleen are recruited to the infarcted area, and experimental evidence demonstrates the importance of MCP-1 and its receptor, CCR2, in the process of leukocytosis [123,124]. The monocyte response to MI proceeds in a biphasic manner whereby distinct monocyte phenotypes are responsible for the expression of CCR2 by damaged cells. MI initiates recruitment of Ly-6C^High^ monocytes from the bone marrow and spleen to the infarcted region. This inflammatory monocyte subtype is highly invasive and is among the first cells to arrive at the infarct zone. Ly-6C^High^ monocyte numbers reach their peak approximately three days after injury [125]. From days 1–3 monocytosis facilitates the influx of monocytes which accumulate and differentiate into macrophages. This is referred to as the inflammatory phase in which macrophages and neutrophils clear dead cells and cell debris. The reparative phase is a result of proper resolution of the inflammatory phase, and is characterized by phenotypic transition of inflammatory monocytes and macrophages (Ly-6C^High^ monocytes and M1 macrophages) to the anti-inflammatory subtypes of the cells (Ly-6C^Low^ monocytes and M2 macrophages). Over the ensuing days fibroblasts proliferate contributing to scar formation. Revascularization of tissues is initiated by monocyte/macrophage production of vascular endothelial growth factor (VEGF) and TGF-β [96,125]. Disfunction of either phase can have profoundly deleterious effects and understanding the mechanics of these elegantly orchestrated processes is essential to the development of novel therapeutic interventions.

As early as 30 min after MI, Ly-6C^High^ monocytes are recruited from the circulation and infiltrate the infarct zone via MCP-1/CCR2 interactions [126]. Monocyte numbers reach their peak at around day three at which point they accumulate and undergo differentiation into macrophages. Determination between monocytes and macrophages in mouse models is achieved by the differential expression of F4/80/I-A^b^/CD11c as well as CD64 and MerTK. Monocytes are classified as F4/80/I-A^b^/CD11c^Low^ and express only CD64, while macrophages are F4/80/I-A^b^/CD11c^High^ and express both MerTK and CD64 [91,125,127]. Between days 5–7 macrophage populations within the infarct are at their maximum as illustrated by a mouse model of MI [128,129]. At this stage, classically activated macrophages dominates the cell population of the infarcted area. M1 macrophages secrete pro-inflammatory cytokines like TNF-α, IL-1β, IL-6 and MMPs, thereby enhancing the pro-inflammatory response and facilitating breakdown of collagen and ECM. It is postulated that pro-inflammatory macrophages play an integral role in preparing the infarcted tissue for repair by removing apoptotic immune cells, dead monocytes, cell debris, and ECM components [130]. The post-MI inflammatory phase gradually subsides and macrophages are again a focal cell type in the process of wound healing. Phagocytosis by macrophages is required for proper initiation of the post-MI wound healing response [131]. Inadequate suppression or extended duration of the pro-inflammatory response that occurs after MI can have serious pathological consequences that include: Cardiomyocyte death, impaired systolic function, chamber dilation due to extensive matrix degradation, compromised ventricular wall integrity, cardiac rupture, and fibrosis [132].

## 10. Macrophages and Tissue Repair after MI

The proliferative phase after MI is demarcated by a shift in the polarization profile of macrophages in the infarcted zone from M1 to M2 phenotype. Depletion of cardiac macrophages with clodronate showed that both M1 and M2 macrophage phenotypes are present in the infarcted area and contribute to tissue remodeling, albeit with divergent roles and effects [128]. A second “wave” of monocytosis takes place once the initial inflammatory phase recedes. The pool of cardiac macrophages is replenished as Ly-6C^Low^ monocytes are extensively recruited to infarcted tissues where they accumulate and undergo differentiation into alternatively activated macrophages. M2 macrophages secrete IL-10, which depresses the pro-inflammatory effects of M1 macrophages, and TGF-β; a cytokine that initiates tissue remodeling and angiogenesis. Shirishi et al. utilized a mouse model with a deletion of the Trib1 gene. This model of impaired M2 macrophage activation allowed them to determine cell type-specific effects of M2 macrophage depletion on post infarct remodeling. When compared to control animals, Trib^-/-^ mice exhibited severely diminished reparative function following MI, including: Reduced collagen synthesis and resultant cardiac rupture. The impaired healing process was completely rescued by the introduction of exogenous M2 macrophages. Furthermore, it was shown that administration of IL-4, which is a well-known activator of M2 macrophages also rescued the dysfunctional genotype [133]. Macrophage heterogeneity in the post-MI heart has profound implications on its ability to promote healing and retain functionality. 

## 11. Macrophage-Targeted Pharmaceutical Interventions

Statins are a class of drugs that inhibit the liver enzyme β-hydroxy β-methylglutaryl-coenzyme A (HMG-CoA) reductase. This effect is designed to reduce the development of atherosclerotic plaque and protect from repeat infarction [134]. Aside from there lipid lowering qualities, statins have exhibited anti-thrombotic and anti-inflammatory properties [135]. Kwak et al. found that pravastatin inhibited IFN-γ-induced macrophage activation [136]. It was also demonstrated by Ghittoni et al. that simvastatin interrupted MHC class II interactions between macrophages and the adaptive immune complex [137].

ACE inhibitors (ACEi) have also been highlighted as possible cardioprotective pharmaceutical therapies. While it’s original usage was prescribed to inhibit circulating angiotensin II (ATII) to concentration, it also mitigates the pro-inflammatory effects of ATII [138,139,140]. In murine mouse models, enalapril reduced ATII-stimulated monocyte recruitment from splenic reservoirs into the myocardium during MI and subsequently improved ejection fraction by 14%, while at the same time mitigating vascular inflammation [141]. In a separate study, enalapril administered after MI resulted in significant reduction in plasma levels of MCP-1 and reduced accumulation and activation of both monocytes and macrophages in patients with MI [142].

## 12. Cell-Mediated Cardioprotection in MI

As mentioned earlier in this review, yolk sac-derived, MHC-IIlowCCR2- and MHC-IIhighCCR2- macrophages are the predominant immune cell type in the healthy heart. This unique population of macrophages decreases with aging and after MI, when they are replaced by the bone marrow derived macrophages (BMDM) via the circulation. This subset of resident, cardiac-tissue macrophages resembles the M2 phenotype and displays cardioprotective attributes post-MI [133]. Although proper infarct healing involves the formation of a functional scar, it has been observed in murine neonates that the distinct population of resident embryonic macrophages is capable of complete restorative repair [96,143]. Newly published work by Dick et al. revealed the importance of resident cardiac macrophages in proper remodeling. [144] The authors utilized a Cxcr1-based method of macrophage depletion coupled with a green fluorescent protein (GFP) label that enabled specific phenotype selection and cell mapping. Despite accounting for only 2–5% of the cardiac macrophage population during the initial weeks post MI, depletion of the cells prevented normal infarct healing and adversely affected cardiac function. And while recruited macrophages were nearly identical to the depleted resident macrophages, they were unable to adopt the same phenotypic specificity [144]. Clearly, resident cardiac macrophages are a novel method of cell-mediated cardio protection. Further work is necessary to better understand what clinical applications these cells may hold. 

## 13. Cardioprotection through Cellular Post-Conditioning

The well-regulated involvement of macrophages in each phase of MI suggests that interactions with other cell types affect macrophage behavior. In another example of cell therapy for MI, investigators have found that paracrine signaling from cardiosphere-derived cells (CDC) modulate macrophage activation promoting a phenotypic switch from M1 to M2 [145,146]. CDCs are essentially cardiac mesenchymal stem cells. Moreover, they exhibit clonogenicity and the potential for multi-lineage differentiation [147]. Multiple animal models have been used to examine the efficacy of CDCs in cellular post-conditioning as a means of ameliorating MI-associated pathology [148]. Intra-coronary infusion of CDCs resulted in decreased infarct size and reduced microvascular occlusion after 48 h in a porcine model [149]. Similarly, a mechanistic study performed in rats with acute MI found that intracoronary infusion of CDCs after 20 min of reperfusion resulted in diminished infarct size and a greater degree of functional recovery. Further, these authors showed that CDCs promoted the alternative activation of macrophages toward a cardioprotective phenotype. Macrophages preconditioned with CDCs were also adoptively transferred after reperfusion and effectively reduced infarct size. Thus, illustrating the interplay between various cells involved in cardiac preconditioning [146]. While the relationship between CDCs and macrophages has only recently been studied, the efficacy and safety of CDCs has been well documented in animal and human subjects [150,151,152,153]. Cellular post-conditioning is especially attractive because of its ability to be administered after an MI event has occurred. Encouraging results and a growing breadth of insight regarding CDCs and macrophages hold much promise for this novel treatment.

## 14. Concluding Remarks

**Figure 1:**
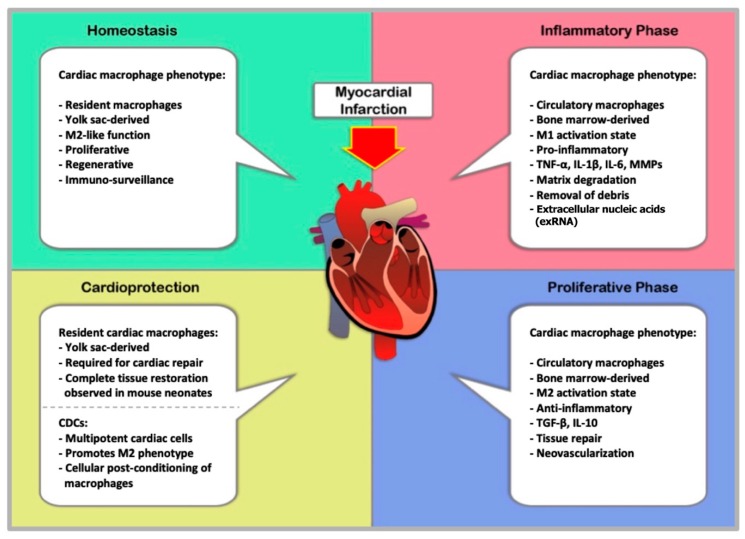
The Cardiac macrophage population, while the heart is in homeostasis, consists primarily of yolk sac-derived macrophages. However, these resident tissue macrophages diminish in number with age and are replaced by circulating bone marrow-derived monocytes even in the absence of inflammation. BMDM replenish the cardiac macrophage supply, and exhibit M2 activation in the steady-state heart. The inflammatory phase of acute myocardial infarction initiates an immune response by monocytes/macrophages, which are recruited to the injured tissue. Ly6CHigh monocytes infiltrate and accumulate in the injured area where they differentiate into “classical” M1 macrophages. M1 macrophages secrete pro-inflammatory cytokines and MMPs upon activation by DAMPs. The inflammatory response peaks at around day 5–7 and ultimately results in clearance of dead cells and cell debris, processes essential for tissue repair. Resolution of inflammation after MI marks the beginning of a proliferative phase. During which, circulating Ly6CLow monocytes infiltrate the infarcted area. Thus, the population of cardiac macrophages begins to move toward alternative activation. The presence of M2 macrophages suppresses inflammation, enhances tissue repair and promotes angiogenesis by the expression of TGF-β and IL-10. Dysregulation of either the inflammatory or proliferative phase can produce severe outcomes like ventricular weakening, fibrosis, and compromised function. Macrophages can participate in cardioprotection via cell-mediated mechanisms. For example, yolk sac-derived cardiac macrophages are required for reparative mechanisms of wound healing. Further, the identical phenotype in murine neonates has full regenerative capacity after MI and remains through adulthood although it diminishes greatly over time and post-MI. CDCs are another example of cellular cardioprotection. The cardiac type of mesenchymal stem cell, CDCs have the potential to induce M2 activation in macrophages. Post-conditioning of macrophages with CDCs results in an alternatively activated phenotype, which has been shown to improve prognosis following myocardial infarction. Abbreviations: BMDM; bone marrow derived macrophages, DAMP; damage associated molecular pattern, MMP; metallo-matrix proteinase; IL-10; interleukin-10, TGF-β; transforming growth factor-β, CDC; cardiosphere-derived cells.

The dynamic mechanisms that govern tissue repair and wound healing are inextricably intertwined with those that mediate inflammation and immunity. The data reviewed here describes a tightly regulated sequence of events from infarction to remodeling that is dependent on different populations of macrophage phenotypes throughout the various stages of myocardial infarction, as summarized in Figure 1. Both M1 and M2 macrophages are required for healing cardiac tissue and preserving functional architecture. Cardioprotection is an intriguing therapeutic technique. However, numerous publications have exposed the elusive nature of this concept. Recent studies are reintroducing much-needed enthusiasm into understanding the cardioprotective potential of cardiac macrophages. As insight beckons effort and innovation, it is imperative that we expand our comprehension of macrophage contributions to cardiac health.

## Abbreviations

CVD	Cardiovascular diseases
DALYs	Disability adjusted life-years
MI	Myocardial infarction
PE	Pulmonary embolism
LDL	Low-density lipoprotein
ROS	Reactive oxygen species
ER	Endoplasmic reticulum
MMPs	Matrix metalloproteinases
ECM	Extracellular matrix
CXCLs	Chemokine (C-X-C motif) ligands
CCLs	Chemokine ligands
MIP	Macrophage Inflammatory Proteins
RANTES	Regulated on Activation, Normal T Cell Expressed and Secreted – CCL5
MCP	Monocyte chemoattractant protein
PAMPs	Pathogen-associated molecular patterns
DAMPs	Damage-associated molecular patterns
PRRs	Pattern recognition receptors
TLRs	Toll-like receptors
MYD88	Myeloid differentiation primary response 88
IRAK-4	Interleukin-1 receptor-associated kinase 4
TRAF	TNF receptor associated factor
IKKβ	IκB kinase β
NF-κB	Nuclear factor-kappa B
ILs	Interleukins
IFN	Interferon
NK	Natural-killer cells
NO	Nitric oxide
SR	Scavenger receptors
MR	Mannose receptors
TGF-β	Transforming growth factor-β
MHC-II	Major histocompatibility complex class II molecules
CCR	Chemokine receptors
ARG 1	Arginase-1
GCR	Glucocorticoid receptor
FN-1	Fibronectin-1
TRIB1	Tribbles homolog 1 is a protein kinase
VEGF	Vascular endothelial growth factor
BMDM	Bone marrow derived macrophages
CDC	Cardiosphere-derived cells
GFP	Green fluorescent protein
